# Symmetry selected quantum dynamics of few electrons in nanopillar transistors

**DOI:** 10.1038/s41598-019-56256-7

**Published:** 2019-12-27

**Authors:** Yue-Min Wan, Heng-Tien Lin

**Affiliations:** Department of Electronic Engineering, I-Shou University, Kaohsiung, Taiwan, ROC

**Keywords:** Materials science, Mathematics and computing, Nanoscience and technology, Physics

## Abstract

Study on single electron tunnel using current-voltage characteristics in nanopillar transistors at 298 K show that the mapping between the N_th_ electron excited in the central box ∼8.5 × 8.5 × 3 nm^3^ and the N_th_ tunnel peak is not in the one-to-one correspondence to suggest that the total number N of electrons is not the best quantum number for characterizing the quality of single electron tunnel in a three-dimensional quantum box transistor. Instead, we find that the best number is the sub-quantum number n_z_ of the conduction z channel. When the number of electrons in n_z_ is charged to be even and the number of electrons excited in the n_x_ and n_y_ are also even at two, the adding of the third electron into the easy n_x_/n_y_ channels creates a weak symmetry breaking in the parity conserved x-y plane to assist the indirect tunnel of electrons. A comprehensive model that incorporates the interactions of electron-electron, spin-spin, electron-phonon, and electron-hole is proposed to explain how the excited even electrons can be stabilized in the electric-field driving channel. Quantum selection rules with hierarchy for the n_*i*_ (*i* = x, y, z) and N = Σn_*i*_ are tabulated to prove the superiority of n_z_ over N.

## Introduction

Single electron tunnel (SET)^1^ is an inspiring topic in quantum box transistors (QBTs)^2,3^ owing to its diverse applications in single-electron devices such as digital memory cells, high precision electro-mechanical sensors and standardized meters^4,5^ that consume one-tenth of pico-watt energy to upgrade the science and technology of semiconductors. With the application of a fixed, small bias on the drain-source electrodes and adjusting the bias on the gate electrode in a QBT, single electron tunnel peaks in current-voltage *(I–V)* characteristics can be manifested at a broad temperature range from milli Kelvin to 300 K. These peaks have been used to disclosure striking electro-quantum-mechanical effects which are known as Coulomb blockade^6^, Coulomb-blockade oscillations^7,8^, Coulomb staircases^9,10^, single electron charging^11–13^, zero-dimension states^14–16^, competing channels^17^, and two-level resonances^18–23^ in various materials^24,25^.

Theoretical understanding of these effects are based on two considerations. One relies on the classical charging energy E_c_ = e^2^/2 C of multiple electrons in a parallel-plate capacitor that has been unanimously confirmed in constant peak spacings. The other depends on size quantization that appears to be more complicated because of the geometrical anisotropy in a three-dimensional (3D) QB. This property can lead to multiple charging energies E_n_ which will modulate peak’s amplitudes^26^ and spacings. Luckily enough, when the geometrical symmetry is introduced into the 3D QBT, the ordering of E_n_ can be brought out and reflects on the sub-quantum number n_*i*_ where *i* denotes the three independent variables x, y, z in a Cartesian coordinate; z, r, θ in a cylindrical coordinate and r, θ, ϕ in a spherical coordinate. Based on the circular symmetry in a two-dimensional (2D) electron gases, Tarucha *et al*. performed a study^27,28^ on a disk QBT in zero magnetic field and found the one-to-one correspondence between the ionization energy of the N_th_ electron discharging from the N_th_ tunnel peak to uncover two magic quantum series. The first is all even numbers of 2, 6, and 12 claimed to be the full filling of electrons in the first, second, and third shells in a two-dimensional (2D) harmonic potential; the second is mixture of even and odd numbers at 4, 9, and 16 being claimed the half filling in the second, third and fourth shells to mimic three-dimensional (3D) spherically symmetric shells of 1*s*, 2*s*, 2*p*, 3*s*, 3*p*, etc. in real atoms to demonstrate the existence of artificial atoms.

These outstanding numbers no doubt prove the effectiveness of Pauli exclusion principle for spin-spin alignments between electrons and of Hund’s rule for charge-charge interactions among electrons in different channels. However, the dynamical symmetry of n_*i*_ electrons are not explored, leading to the paucity of understanding how single electron tunnel is generated in the QBT. In our view, the N electrons will not be self-confined in the 2D x-y plane although they were assumed to be frozen at a very low temperature ∼50 mK^26^. Some of the electrons ought to be excited and stabilized in the conduction channel and that will make the interactions between the electrons in the x-y channels and those in the z channel critical which are believed to be the key of understanding the dynamical effects of SET in a deeper level; so does to the aforementioned seven effects.

In this paper, we will demonstrate such picture of n_*i*_ with the focus aiming on how the electrons excited in the narrow conduction z channel can be stabilized firstly, then on how they interact with the n_x_ and n_y_ electrons. It is found that the interactions of electron-electron, spin-spin, electron-phonon and electron-hole (exciton) in the z channel are vital in helping n_z_ becomes the most decisive quantum number. The electrons of n_x_ and n_y_ collaborate with the mechanical vibrations to assist the indirect tunnel of single electron. Each n_*i*_ is found to obey the fundamental rules of even number in doing electro-quantum mechanical stability and the odd number is in doing instability. As a harmony effect, the first peak generated in the stabilized SET is triggered by the addition of the third electron into the easy x and y channels when two (or four) electrons are pre-excited in the z channel. With all the n_*i*_ being assigned to all the peaks in the *I-V* spectrum, a comprehensive model is established to map out a full chart of n_*i*_ and N that eventually leads to the conclusion that n_z_ is the principle quantum number in characterizing the transport quality of single electron tunnel.

## Mirror Symmetry of Moving Electrons in a Box

Once the importance of the dynamical symmetry is introduced^29^, its influential effects must be established based on the degrees of balance and stability in a multiple electrons system by checking whether the moving electrons obey the symmetry of mirror images. When two electrons with antiparallel spins^30^ and equally apart from the box’s center move toward (or away from) each other in a one-dimensional well, the symmetry is called conserved; whereas when the condition is of one moving electron only, the symmetry has no chance to be conserved. Extending such an idea to 3D, the total N acting electrons and the sub acting n_*i*_ electrons in each channel clearly are periodical functions of the number 6 and 2, denoted as N_e_ and n_ie_. At these numbers, the box is at the peak of stability. While at the middle odd numbers denoted as N_o_ = 3 and n_io_ = 1, the box is at the valley of stability. Assuming the N is increased at the steady pace of +1 for each tunnel peak, the N_e_ and n_ie_, N_o_ and n_i0_ will alternatively play conservation and breaking of the global-3D and local-1D symmetries. In accordance, the N_e_ comes as the series of 0, 6, 12, 18…; the N_o_ as 3, 9, 15, 21…; the n_ie_ as 0, 2, 4, 6… and the n_io_ as 1, 3, 5, 7…etc. The combination of N_e_ and n_ie_ is possible and it becomes the series of 2, 8, 14, 20…; the N_e_ + n_io_ of 1, 7, 13, 19…; the N_o_ + n_ie_ of 5, 11, 17, 23… and the N_o_ + n_io_ of 4, 10, 16, 22…etc. The combination of n_ie_ + n_io_ no doubt will become the most flexible and popular states. These values will be confirmed later.

**Figure 1:**
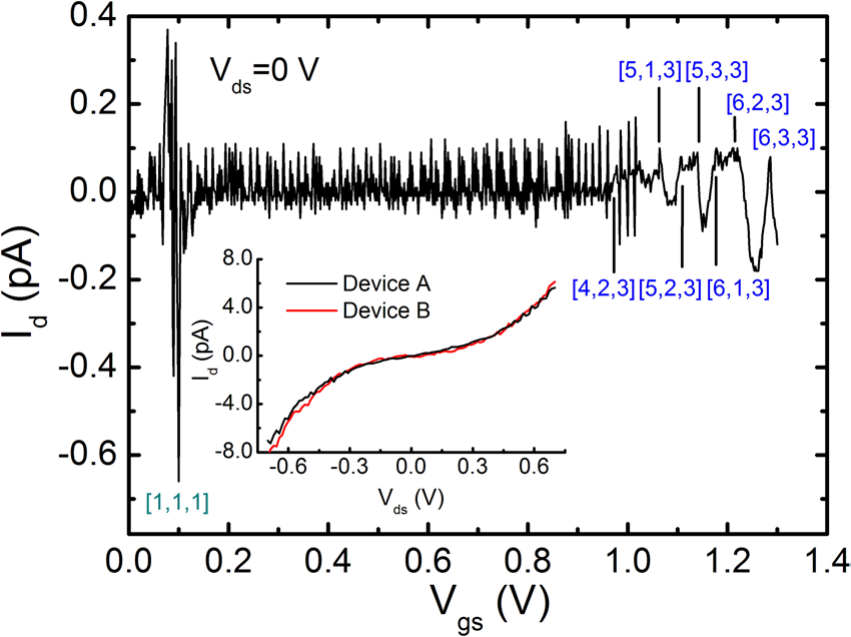
I_d_ versus V_gs_ of device B at 298 K. Giant Coulomb currents are clear near 0.1 V. The leading 3D electronic states of n_z_ = 3 dominate the tunnel peaks at high bias. Inset shows Coulomb blockades of device A and B as fabricated.

Transport measurements are performed on nanopillar transistors. The QB size in the devices is ∼8.5 × 8.5 × 3 nm^3^ that makes the lateral to normal length ratio 3 to 1, better than the disk QB of 8 to 1. The method adopted to analyze the *I*_*d*_ − *V*_*ds*_ characteristics is unprecedented. Both the N and the n_*i*_ of each peak are determined. With these information, the total electronic numbers determined are up to n_z_ = 4 and N = 28. Given the sizes, Eq. (1) is used to calculate all the possible single-particle states, denoted as [n_x_, n_y_, n_z_], along with the associated energies E_n_ within 1 eV^31^. Table 1 summarizes all of them. Devices A and B were selected for discussions. Their qualities were checked by the as-fabricated *I-V* plots shown in the inset of Fig. 1. Both curves clearly exhibited symmetric effects of Coulomb blockades with CV_ds_ = e at V_ds_ ≤ ±0.13 V of I_d_ = 0 A. The value of C was determined to be ∼1.2 aF which exactly matched the net value of the SiN_x_-Si-SiN_x_ trinature frequency and-capacitors in demonstrating their high qualities.

1 $$E({n}_{x},{n}_{y},{n}_{z})=\frac{{\pi }^{2}{\hslash }^{2}}{2m}(\frac{{n}_{x}^{2}}{{L}^{2}}+\frac{{n}_{y}^{2}}{{W}^{2}}+\frac{{n}_{z}^{2}}{{H}^{2}})$$

**Table 1:**
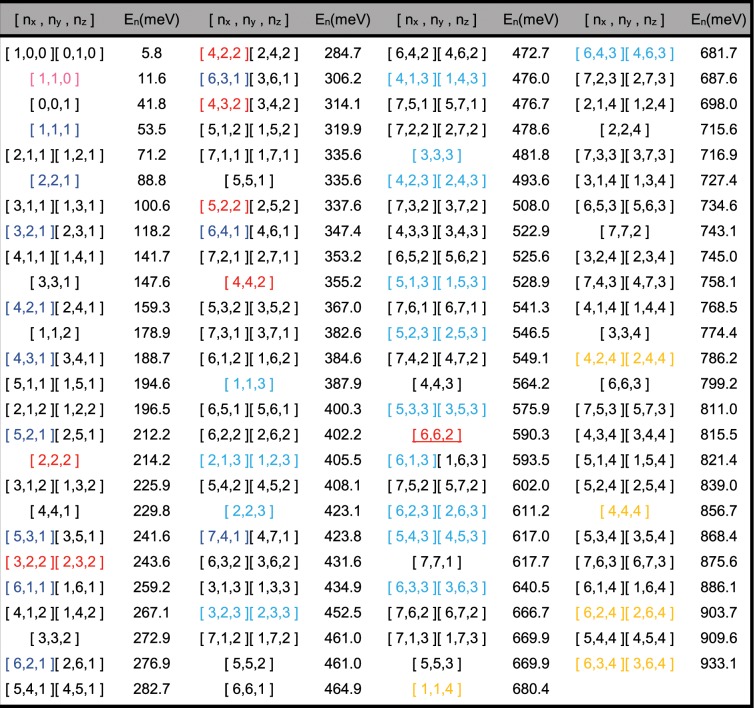
Three-dimensional quantized states and energies of a 8 × 8 × 3 nm^3^ box.

The data are calculated from Eq. (1) (see main text). The states in magenta, dark cyan, red, blue and orange colors correspond to those of n_z_ = 0, 1, 2, 3 and 4. It is evident that in the highly symmetric device B, the number of states selected is very few ∼13, while in the less symmetric device A, the states selected are vast, more than 40.

## Results and Discussions

### Coulomb-blockade oscillations in nz = 0

**Figure 2:**
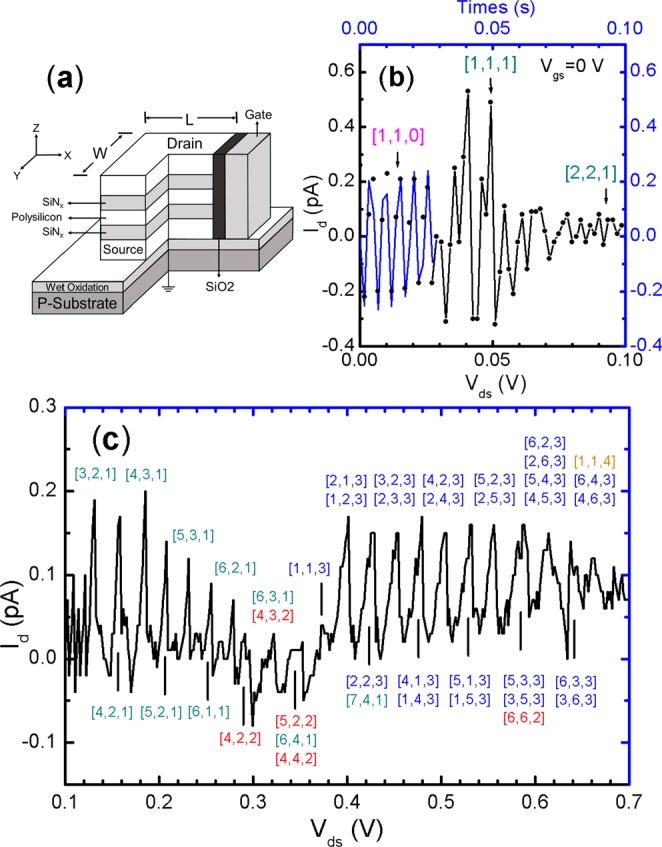
Device structure and current-voltage characteristics of device A at 298 K. **(a)** A schematic view of nanopillar transistor. (**b)** A theoretical fit and data. The blue curve is the damping current calculated from Eq. (2) (see main text). Parameters used in the fit are n = 5 × 10^22^/cm^3^, A = 72 nm^2^, α = 3 Å, ω = 1.16 × 10^3^ Hz, φ = π/2 and ξ = 6 × 10^−3^. (**c)** *I*_*d*_*–V*_*ds*_ at large bias. The 3D electronic states of each tunnel peak are labeled in brackets, referring to Table 1. Odd peaks are labeled on top and even peaks are below. The first series of single-electron tunnel at n_z_ = 1 is in dark cyan, the second series of n_z_ = 3 is in blue. The states at n_z_ = 2 and 4 of stop zones are in red and orange.

As shown in Fig. 2(b), the first two electrons of the [1, 1, 0] state generate a moderate electron-phonon interaction by yielding Coulomb vibrations and subsequent currents. The alternating currents at V_ds_ < 30 mV suggest that the n_x_ and n_y_ electrons which are scattering created from the *Electric*-field in the z-channel. Their circular motions are dissipative and force free that can be described by the classical equation of motion with the induced current^32^ as

2 $${\rm{I}}({\rm{t}})=neA\alpha \omega {e}^{-\xi {\omega }_{n}t}\,\cos (\omega t+\varphi )$$

where n is silicon density, A box area, α maximum displacement, 𝜉 damping ratio c/c_c_, c_c_ = 2 mω_n_, ω = ω_n_(1 − ξ^2^)^1/2^, ω_n_ = (k/m)^1/2^ nature frequency and φ the initial phase. The good numerical fit to data in t ≤ 0.3 s proves that the paired electrons are indeed doing harmonic motions and the phase φ is also determined to be π/2.

**Figure 3:**
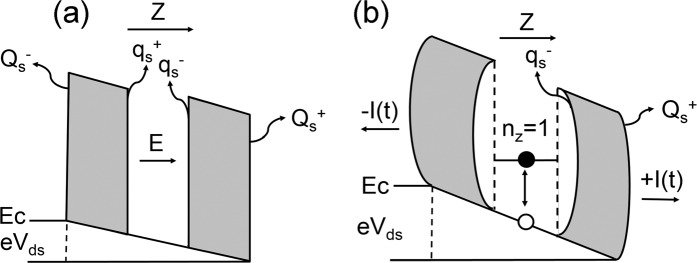
Dielectric response under the action of a drain-source voltage. (**a)** An electric field *E* is initiated inside of the box with accumulated surface charges Q_s_^−^, q_s_^+^, q_s_^−^ and Q_s_^+^. **(b)** Single electron excited to state n_z_ = 1 and box deformations. A hole is also created. Coulomb forces between electron and q_s_^−^ and q_s_^+^ generate forward +I(t) and backward −I(t) currents.

When the third electron is added to the QB for the state [1, 1, 1]. The dynamics of n_z_ = 1 electron show up. As illustrated in Fig. 3(a,b), the *E*-field generated free electron can lead to significant deformations and mechanical feedbacks in the QBT^33^. At the very first moment of a small bias being applied onto the box, as shown in Fig. 3(a), surface charges of positive and negative polarities will be built up on all interfaces^34^, denoted as Q_s_ and q_s,_ respectively. When the eV_ds_ reaches E(n_z_ = 1) ∼42 meV, the electron generates a large Coulomb force to bend the box into ±z directions sequentially as illustrated in Fig. 3(b). Note that the initial repulsive force is on the q_s_^−^, and it will then result in a forward current +I(t). The elasticity of the QB will restore it to its initial position, and excite the hole to provide a counterforce on the other end of the QB for a backward current −I(t). As results, zig-zag currents^35,36^ are created between [1, 1, 0] and [1, 1, 1] states.

### Unstable single electron tunnel in nz = 1, 3

**Figure 4:**
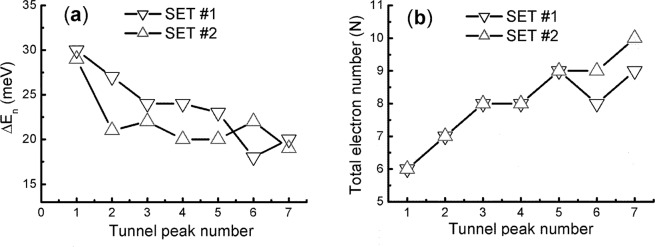
Single electron tunnel peak dependence of charging energy and total electron number N. (**a)** Peak number versus charging energy. The first point represents the energy gap between [2, 2, 1] and [3, 2, 1] states. The remaining points represent the energy spacing between two neighboring peaks in Fig. 2(c). (**b)** Peak number versus N. The instability of N is clear as it comes across the number of 9.

At the state [3, 2, 1] of six electrons, the first SET shows up in Fig. 2(c) and it is triggered by the addition of the n_x_ = n_y_ = 3 electron into the [2, 2, 1] state (see Table 1). After that, the first series (#1) of SET develops as the N is increased from 6 to 9, [6, 2, 1], for a total of seven sharp peaks. Each peak is clean in singlet state, suggesting a unique vibration mode is involved, and they are separated by a seemingly constant distance. For the QB of A ≈8.5 × 8.5 nm^2^ and H = 3 nm, the E_c_ = e^2^/2 C, C = ε_r_ε_o_A/D ∼3.5 aF, ε_r_ = 11.7, ε_o_ = 8.85 × 10^−12^ C^2^/Nm^2^ is estimated to be ∼30 meV. This value is expected to match the peak spacing ΔE_n_ well and as matter of fact, in Fig. 4(a), the ΔE_n_ varies strongly from peak to peak with the 1^st^ ∼30 meV and the 6^th^ ∼18 meV that yields a statistical value ∼24 ± 6 meV and a mean deviation over 20%. The large deviation indicates that the n_z_ = 1 SET of is not stable, with spin 1/2. As a consequence, the SET stops at the states [4, 2, 2]/[2, 4, 2] and [4, 4, 2] of n_z_ = 2 which have s = 0.

Notably, at a higher eV_ds_ ∼0.4 eV, the stopped SET revives itself by changing from the singlet to doublet state (meaning L ≠ W), and also by regrouping the n_i_ to increase the n_z_ from 1 to 3 while maintaining the total N fixed at 6. In the following 10 split peaks, the N quickly increases from 12 to 24. Thanks to the extra stability introduced by the doublet states^17^, the #2 SET runs a little bit longer than the #1. Accordingly, the distributions of ΔE_n_ becomes a uniform value of ∼25 ± 3 meV in Fig. 4(a). Nevertheless, these peaks still show progressively decays in amplitudes for another end near 0.7 V to prove that the odd n_z_ = 3 is still unstable (s = 1/2).

### Stable single electron tunnel in nz = 2, 4

**Figure 5:**
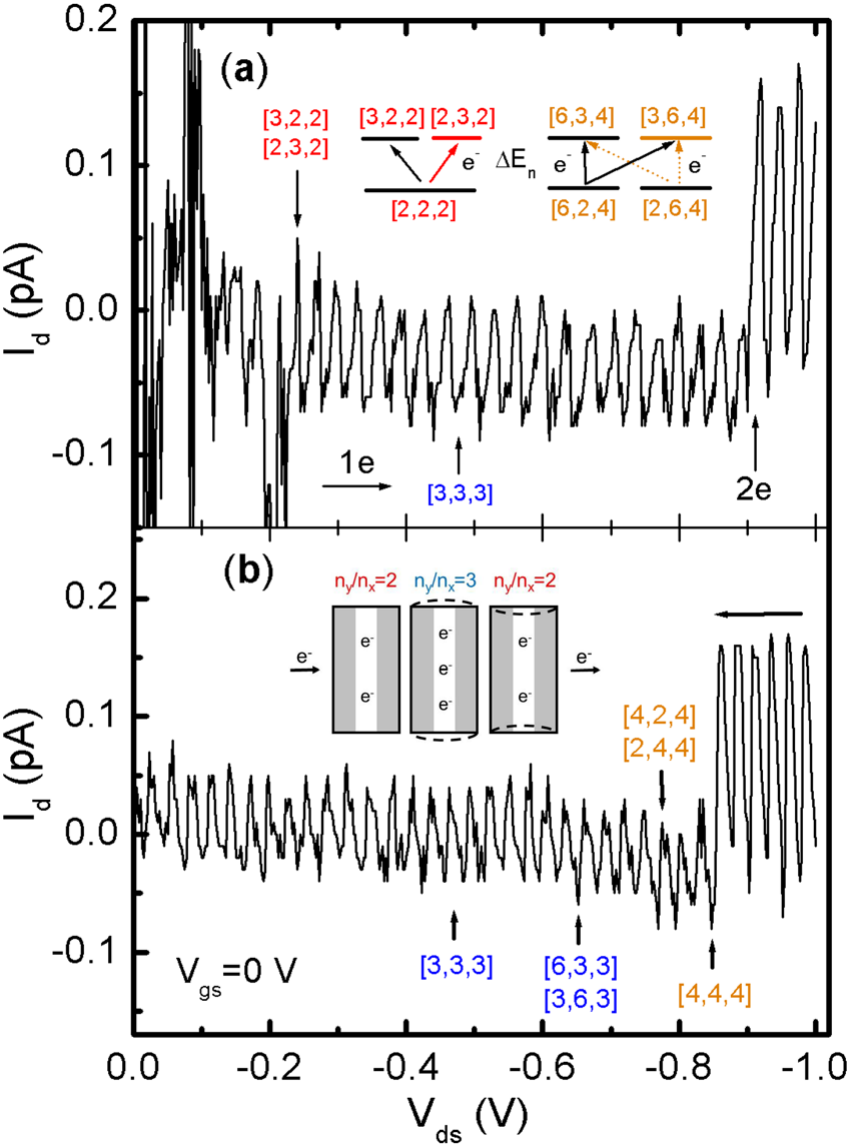
I_d_ versus V_ds_ of device B at 298 K. Note that the polarity of current is changed. (**a)** Electron charging from 0 to 1 V. The first peak is at [3, 2, 2]/[2, 3, 2] doublet. Double electron tunnel begins at [6, 2, 4]/[2, 6, 4] states. Inset (left) shows two-level resonance of one electron tunnel; (right) two-channel, two-level resonance tunnels of double electrons. (**b)** Reversed charging from −1 to 0 V. Transition from 2*e* to 1*e* tunnel is at [4, 4, 4] state. Inset shows the vibration assisted electron tunnel; from left to right, prior to the entrance of 1*e*, resonances with 2*e* in the n_y_/n_x_ channels, and repel of the charged electron.

To make the n_z_ = 2 SET happen, device B is used. In Fig. 5(a), it is again that giant Coulomb currents dominate the *I*_*d*_–*V*_*ds*_ from 0 to −0.23 V. The first peak emerges right at the doublet [3, 2, 2]/[2, 3, 2] states to confirm that the starting n_z_ is indeed 2^14^. After that the SET peaks are well spaced by a ΔE_n_ which is estimated to be ∼33 ± 3 meV. This value matches the E_c_ ≈34 meV very well. By checking with Table 1, the ground state of all the two-level resonances is at the most balanced N_e_ = 6, [2, 2, 2] state. The gap ΔE_n_ ∼29.4 meV between [2, 2, 2] and [3, 2, 2]/[2, 3, 3] is an excellent value near E_c_. No wonder that the stable SET runs very long for a total of 21 doublet peaks^24^. In fact, they are triggered by the N = N_e_ + n_io_ = 6 + 1 = 7 electron in each mode with 2 N = 14 in total. *In another words, it is the third electron added to the stable n*_*x*_ = *n*_*y*_ = *2 states creating the weak dynamical break to assist one electron tunnel in the z-channel*. Inset of Fig. 5(b) illustrates how this n_x_/n_y_ = 3 electron can take advantage of the two-level vibrations^18–23^; it is firstly charged into the n_x_/n_y_ channels, then propelled out of them into the main channel for a current peak.

At eV_ds_ ∼ −0.92 eV, the SET makes a transition to double electron tunnel (DET) by presenting a sharp increase in the magnitude of I_d_ that resembles the Coulomb staircases which have been spotted elsewhere^9,10^. The cause of this transition is created by the same ΔE_n_ ∼29.4 meV which is amazing. Notice that although the DET occurs at the next even n_z_ = 4, the same n_x_/n_y_ = 2 ↔ 3 resonance remains active. Ideally, the resonance should happen at the global ground state of [4, 4, 4] (just like [2, 2, 2]) and then jump to the higher states of [5, 4, 4]/[4, 5, 4], but that would require an much higher energy ∼53 meV which is almost impossible. As an alternation, the QBT skips three energy levels until the meet of the doublet ground states [6, 2, 4]/[2, 6, 4]; therein the QBT makes the transition to the doublet [6, 3, 4]/[3, 6, 4] states. As a reward, one more conduction channel is created, as shown in the inset of Fig. 5(a), making electrical conduction more efficient. In the meantime, the total N reaches the highest value of 4N_e_ + 4n_o_ = 28.

The robust SET and DET are reproducible from a high bias. In Fig. 5(b), when the bias begins with the −1 V, the DET shows no sign of fading at all in the beginning. The EQM coherence remains firmly locked with the N = 24 ↔ 28 transitions, with the only difference being the φ shifted from the π/2 (t = 0 s) to π. At the all even [4, 4, 4] state, the DET makes a decay to the SET. After that, the N = 12 ↔ 14 resonances take over and run the SET all of the way to V_ds_ = 0 V to convince the initial Coulomb-blockade oscillations observed in Fig. 2(b). These data also explain the long-time search of why the classical E_c_ is an excellent indication of stable electron charging energy and it is only when the E_n_ matches its value, the coherent SET will show up. Other than the outstanding finding, the breakdown of the one-to-one correspondence between the N and the tunnel peaks is also remarkable as it indicates that there will have multiple peaks in corresponding to the same electron numbers and their effects will be discussed in the following paragraph.

### Excitation of holes to neutralize electrons

The transition from DET to SET drastically changes the value of N. Here, the ΔN is as large as 16, from 28 to 12 at the state of [4, 4, 4] that makes 16 excited electrons left over to be annihilated by holes^37^. As results of the holes excitations illustrated in Fig. 3(b), negative mechanical feedbacks will constantly act on the drain electrode for negative currents to lower the level of I_d_. As a consequence, after the largest drop at [4, 4, 4], the next drop develops at the doublet [4, 2, 4]/[2, 4, 4], then is the hidden n_z_ = 3 [6, 3, 3]/[3, 6, 3] doublet states, following by the all odd singlet [3, 3, 3] right in the middle of the SET series. Notably, in Fig. 5(a) of electron charging in device A, the [3, 3, 3] state is also becoming visible in a dip to signal that the weakly bonded pairs of electrons and holes (excitons) are important dynamical entities. The same effect is also detected in Fig. 2(c), when the ΔN is not increased in pace with the +∆eV_ds_, three drops appear at the [4, 2, 2], [4, 3, 2]/[3, 4, 2], [4, 4, 2] and [5, 2, 2]/[2, 5, 2] states.

### Interferences in two conduction channels

Electron charging from the side gate confirms the high flexibility of n_ie_ + n_io_. Here the n_ie_ represents the sum of n_xye_ and n_xyo_, and the n_io_ is the n_zo_. The reason is simple because the driving *E*-field will be separated into two components; one is along the side channel and the other is along the main channel. As results of their interference acting^38^, the states excited are most likely to be the highest probability but with the lowest symmetry. As shown in Fig. 1, The first giant currents of [1, 1, 1] again appear at V_gs_ ∼ 0.1 V. When compared to the noise at 50 mV in Fig. 2(b), the gate-dot coupling strength is determined to be 0.5. Based on this value, the successive leading peaks for SET are identified to be the following states; [4, 2, 3], [5, 1, 3], [5, 2, 3], [5, 3, 3], [6, 1, 3], [6, 2, 3] and [6, 3, 3]. Notice that there are no all even states like N_e_, the starting N_o_ is at 3, then is the isotropic state of N_o_ = 9, [4, 2, 3], the N then fluctuates down and up in the states of n_z_ = 3. At the [6, 3, 3], an isotropic state of N = 12, a sharp peak appears in reminding of the end in SET #2.

### Quantum selection rules in 3D box transistors

As listed in Table 2, the hierarchy of symmetry in terms of the N_e_, N_o_, n_ie_, and n_io_ becomes very clear and the result is N_e_ > n_ie_ > n_io_ > N_o_. N_o_ of the lowest stability manifests itself as the giant Coulomb currents in [1, 1, 1] and [3, 3, 3]. The [1, 1, 1] state also serves to activate the QBT for later on tunneling of electrons. In sharp contrast, the N_e_ provides the highest stability in [2, 2, 2] and [4, 4, 4] states for an activated QBT. The primary combination of N_e_ + n_xyo_ then creates the most needed tunneling of single electron. The secondary combination of n_ie_ + n_xyo_ generates the most urgently needed two-channel, two-level resonance tunneling of double electrons. In between, the combination of n_xye_ + n_xyo_ + n_zo_ creates most of the unstable SET and they will be temporarily stopped at the states of n_xye_ + n_xyo_ + n_oe_. According to these rules, the previously claimed *not-all* odd magic number of 4, 9, and 16 in disk QBT could be either the all odds number of 3, 9, 15, or the composition of n_xye_ + n_z_ with the latter being the incremental number of 0, 1, and 2. After subtracting the n_z_, that leaves the n_xye_ = 4, 8, and 14 which fit perfectly into the *claimed* all even numbers of 2, 6, and 12.

**Table 2:** Three-dimensional electronic states identified in device A and B.

	Device A								Device A						
	[n_x_, n_y_, n_z_]	n_z_	N	N_e_	n_ie_	n_io_	N_o_		[n_x_, n_y_, n_z_]	n_z_	N	N_e_	n_ie_	n_io_	N_o_
Charging n_z_ = 0, 1	[1, 1, 0]	0	2		O	O		n_z_ = 3, 4	**[4, 1, 3]**	3	16		O	O	
	[1, 1, 1]	1	3			O			**[4, 2, 3]**	3	18		O	O	
	[2, 2, 1]	1	5		O	O			**[5, 1, 3]**	3	18			O	
	[3, 2, 1]	1	6		O	O			**[5, 2, 3]**	3	20		O	O	
	[4, 2, 1]	1	7		O	O			**[5, 3, 3]**	3	22			O	
	[4, 3, 1]	1	8		O	O			**[6, 2, 3]**	3	22		O	O	
	[5, 2, 1]	1	8		O	O			**[5, 4, 3]**	3	24		O	O	
	[5, 3, 1]	1	9			O			**[6, 3, 3]**	3	24		O	O	
	[6, 1, 1]	1	8		O	O			**[6, 4, 3]**	3	28		O	O	
	[6, 2, 1]	1	9		O	O			[1, 1, 4]	4	6		O	O	
	[6, 3, 1]	1	10		O	O			**Device B**						
	[6, 4, 1]	1	11		O	O			**[n**_**x**_**, n**_**y**_**, n**_**z**_**]**	**n**_**z**_	**N**	**N**_**e**_	**n**_**ie**_	**n**_**io**_	**N**_**o**_
	[7, 4, 1]	1	12		O	O		Charging	[2, 2, 2]	2	6	O	O		
n_z_ = 2, 3	[4, 2, 2]	2	8		O				**[3, 2, 2]**	2	14		O	O	
	[4, 3, 2]	2	9		O	O			[3, 3, 3]	3	9			O	O
	[5, 2, 2]	2	9		O	O			**[6, 2, 4]**	4	24		O		
	[4, 4, 2]	2	10		O				**[6, 3, 4]**	4	28		O	O	
	[6, 6, 2]	2	14		O			Discharging	**[6, 3, 4]**	4	28		O	O	
	[1, 1, 3]	3	5			O			[4, 4, 4]	4	12	O	O		
	**[2, 1, 3]**	3	12		O	O			**[4, 2, 4]**	4	20		O		
	[2, 2, 3]	3	7		O	O			**[6, 3, 3]**	3	24		O	O	
	**[3, 2, 3]**	3	16		O	O			[3, 3, 3]	3	9			O	O

Hierarchy of quantum numbers denoted and classified by N_e_, n_ie_, and n_io_ and N_o_ symbols. N_e_ has the highest symmetry in a state with all even n_i_ and n_x_ = n_y_ = n_z_. n_ie_ comes as the next with at least one even n_i_ in a state, n_io_ is ranked as the third with at least one odd n_i_ in a state. N_o_ is the lowest in ranking with all odd n_i_ and n_x_ = n_y_ = n_z_ in a state. Bold state means doublet states. It is clear that the increment of n_z_ in charging is much steadier than N.

### Vibrational frequencies and maximum displacements

Vibrational frequencies induced by the electron-phonon interactions in the QBTs are estimable. The most outstanding one clearly is the [1, 1, 1] as plotted in Fig. 2(a). Its ν_z_ is fitted to be ∼1.85 × 10^2^ Hz and the maximum displacement of α_z_ is fitted to be ∼3 Å. At N = 7, the values of ν_y_ and ν_x_ are expected to be higher, ∼100 times larger than the initial frequency. Since the C of the two devices is approximate ∼3.5 aF and the R is ∼2 × 10^13^ Ω that leads the RC ∼4.4 × 10^−5^ s and the ν_x_/v_y_ ∼2.2 × 10^4^ Hz. By taking this value into I_d_ = eν_y_, a peak current of 0.15 pA is obtained, which agrees with the data very well. With the vibrating frequency being increased at the n_z_ = 3 states, the α_y_ or α_x_ will become smaller. For an isotropic electron mobility in the QB, the I_d_ equals neAυ_d_ with υ_d_ = αω_y_/2π. Given the n = 10^19^/cm^3^ and A = 64 nm^2^, the α_y_ (or α_x_) is calculated to be ~1 Å.

In conclusion, we study the dynamics of electrons in three-dimensional quantum box, nanopillar transistors. Single electron tunnel N_th_ peak is found not in one-to-one correspondence with the excited N_th_ electron. The best quantum number to describe SET is the sub-quantum number n_z_ in the conduction z channel, not the conventionally used total electron number N. Stable SET peaks occur only when the number of electrons excited in n_z_ is even and the number of electrons excited in the normal n_x_/n_y_ channels are also even at two, robust peaks are then generated one-after-one by the charging/discharging of the third electron in/out of the normal x-y channels. A comprehensive model that incorporates the dynamical interactions of electron-electron, spin-spin, electron-phonon, and electron-hole explains the mechanical stability of even electrons in the electric-field driving z channel. Quantum selection rules with hierarchy of n_*i*_ and N are tabulated to prove that n_z_ is a better number than the N for characterizing the quality of electron tunnel.

## Methods

### Device fabrication and current-voltage measurements

The nanopillar transistor as shown in Fig. 2(a) was fabricated on a p-type (100) silicon wafer which featured a central polysilicon layer separated from the top and bottom electrodes by a nitride layer^39^. This center box had a critical thickness of 3 nm and was coupled to a side gate. Deposition of SiN_x_ (3-nm)-polysilicon (3-nm)-SiN_x_ (3-nm) layers were processed using a low-pressure chemical vapor technique, and then chemically etched it to create a nominal plateau ∼200 × 140 × 210 nm^3^. Phosphorous of a concentration 1 × 10^19^ cm^−3^ was doped in Si layer. The source was located at the bottom with a sheet resistance of ~30 Ω/cm^2^. To prevent electrical shortage, a short oxidation was carried out to seal the nanopillar (creating another ~1.5 nm oxide). To further squeeze the cavity, the technique of self-aligned oxidation was utilized to add another ~6 nm layer of gate oxide, yielding a total of ~9 nm with the inner box reduced to the size ~9 × 9 × 3 nm^3^. Finally, an Al (300 nm) side gate was attached. Devices were loaded into a probe station for *I-V* measurements at 298 K and a three-terminal HP meter with resolutions of 1 mV and 10 fA was used.

### Zero magnetic field

To elucidate the authentic electron charging effects, external perturbations were kept to a minimum. No extra magnetic field was applied, except the negligible *Earth B-*field. Electrical bias was only applied either from the drain-source electrodes or the gate-source electrodes to avoid mutual interference in most cases.

### Reduced sizes in parabolic potential

To count for the mutual interactions between multiple electrons in a parabolic potential, the values of L and W are reduced ∼5% smaller than the real values as the effectively electron-electron repulsions will make the QB smaller for a higher charging energy to the entrance of next electron. With these fine tunings, excellent agreements are reached between analysis and data.
